# Photodynamic N-TiO_2_ Nanoparticle Treatment Induces Controlled ROS-mediated Autophagy and Terminal Differentiation of Leukemia Cells

**DOI:** 10.1038/srep34413

**Published:** 2016-10-04

**Authors:** Mohammad Amin Moosavi, Maryam Sharifi, Soroush Moasses Ghafary, Zahra Mohammadalipour, Alireza Khataee, Marveh Rahmati, Sadaf Hajjaran, Marek J. Łos, Thomas Klonisch, Saeid Ghavami

**Affiliations:** 1 Department of Molecular Medicine, Institute of Medical Biotechnology, National Institute for Genetic Engineering and Biotechnology, Tehran, Iran; 2 Cell and Molecular Biology Department, Pharmaceutical Sciences Branch, Islamic Azad University, Tehran, Iran; 3 Hematology and Oncology Research Center, Tabriz University of Medical Science, Tabriz, Iran; 4 Department of Nanobiotechnology, Faculty of Biological Sciences, University of Tarbiat Modares, Tehran, Iran; 5 Research Laboratory of Advanced Water and Wastewater Treatment Processes, Department of Applied Chemistry, Faculty of Chemistry, University of Tabriz, Tabriz, Iran; 6 Department of Materials Science and Nanotechnology, Near East University, 99138 Nicosia, North Cyprus, Mersin 10, Turkey; 7 Rheumatology Research Center, Tehran University of Medical Sciences, Tehran, Iran; 8 LinkoCare Life Sciences AB, 583 30 Linköping, Sweden; Malopolska Centre of Biotechnology Jagiellonian University Gronostajowa 7A str 30-387 Krakow, Poland; 9 Department of Human Anatomy and Cell Science, College of Medicine, Faculty of Health Sciences, University of Manitoba, Winnipeg, MB, Canada; 10 Children Hospital Research Institute of Manitoba, University of Manitoba, Winnipeg, Canada; 11 Health Research Policy Centre, Shiraz University of Medical Science, Shiraz, Iran

## Abstract

In this study, we used nitrogen-doped titanium dioxide (N-TiO_2_) NPs in conjugation with visible light, and show that both reactive oxygen species (ROS) and autophagy are induced by this novel NP-based photodynamic therapy (PDT) system. While well-dispersed N-TiO_2_ NPs (≤100 μg/ml) were inert, their photo-activation with visible light led to ROS-mediated autophagy in leukemia K562 cells and normal peripheral lymphocytes, and this increased in parallel with increasing NP concentrations and light doses. At a constant light energy (12 J/cm^2^), increasing N-TiO_2_ NP concentrations increased ROS levels to trigger autophagy-dependent megakaryocytic terminal differentiation in K562 cells. By contrast, an ROS challenge induced by high N-TiO_2_ NP concentrations led to autophagy-associated apoptotic cell death. Using chemical autophagy inhibitors (3-methyladenine and Bafilomycin A1), we confirmed that autophagy is required for both terminal differentiation and apoptosis induced by photo-activated N-TiO_2_. Pre-incubation of leukemic cells with ROS scavengers muted the effect of N-TiO_2_ NP-based PDT on cell fate, highlighting the upstream role of ROS in our system. In summary, PDT using N-TiO_2_ NPs provides an effective method of priming autophagy by ROS induction. The capability of photo-activated N-TiO_2_ NPs in obtaining desirable cellular outcomes represents a novel therapeutic strategy of cancer cells.

Nanoparticles (NPs) are particles smaller than 100 nm in size and are of particular interest as cancer therapeutics because they preferentially localize to tumor sites and easily penetrate tissue and cellular barriers. In addition, NPs can be finely-tuned and used for simultaneous therapy and diagnosis (theragnosis)^1^,^2^,^3^. Among NPs, titanium dioxide (TiO_2_) exhibits unique super-photocatalytic properties that can be utilized to kill cancerous cells upon irradiation^2^,^3^,^4^. Under ultraviolet (UV)-light illumination, the valence band electrons of TiO_2_ are excited to the conduction band and the resulting electron holes have the capability of generating various cellular reactive oxygen species (ROS), including hydroxyl radical (OH·), hydrogen peroxide (H_2_O_2_), and superoxide (O2^−^)^4^,^5^. Irradiation-induced generation of ROS by a photosensitizer is called photodynamic therapy (PDT) and has been clinically approved for several diseases, including cancers^6^,^7^. The advantages of PDT compared to other anti-cancer strategies include the lack of known drug resistance and the ability to control ROS production in cancer cells by controlling PDT doses^6^,^7^,^8^. The successful use of TiO_2_ NPs in PDT has been reported for many different types of cancers, such as human cervical adenocarcinoma, hepatocarcinoma, non-small cell lung cancer, and leukemia^5^,^9^,^10^,^11^,^12^. However, the biggest obstacle in the clinical application of TiO_2_-based NPs for PDT is the TiO_2_ high band-gap energy level (3.2 ev for anatase) that requires excitation with harmful UV radiation (λ < 387 nm)^4^,^13^,^14^,^15^. Doping TiO_2_ with metals and/or non-metals usually solves this problem and shifts the absorption onset of TiO_2_ to longer non-toxic wavelengths^13^,^14^,^15^. For example, nitrogen-doping (N-TiO_2_) shifts the absorption range of TiO_2_ to longer wavelengths and leads to a remarkable photocatalytic activity under visible light^16^,^17^,^18^. As an improved nano-photosensitizer, N-TiO_2_ exhibits significant advantages over TiO_2_ with higher ROS-producing capacity and anti-cancer PDT activity, but its mechanism of action has yet to be fully elucidated^18^,^19^,^20^.

Autophagy is a highly conserved process that occurs in response to a variety of stressful conditions and can lead to cell survival and differentiation or cell death, depending on the cellular context and level and type of stress^21^,^22^,^23^,^24^. At the initial steps of this catalytic pathway, large biomacromolecules and/or organelles are sequestered inside of autophagosomes, which fuse with lysosomes to form acidic vesicular organelles (AVOs) and ultimately lead to recycling or degradation of its content^21^,^22^,^23^,^25^. The link between autophagy and cancer is complex. Autophagy can act as tumor suppressor and/or tumor promoter with the outcome depending on disease stage^21^,^23^. Thus, the blockade and induction of autophagy are both exploited in cancer therapies^23^,^26^,^27^. Hence, drug discovery research currently focuses on the identification of autophagy modulators^23^,^26^. Recently, a variety of different NPs, including TiO_2_^9^,^28^, ceria^29^, iron oxide^30^,^31^, rare earth oxides^32^, and carbon nanotubes^33^, effectively induced autophagy and this was mainly dependent on their physicochemical properties (e.g., dispersing state and size) and subcellular sites of NPs accumulation^31^,^32^,^33^,^34^. Detection of NPs inside autophagosomes suggests the initiation of a cellular mechanism aimed at activating autophagy to degrade the internalized NPs^35^,^36^,^37^. However, oxidative stress pathways (e.g., mitochondrial damage and/or endoplasmic reticulum stress) or alteration of expression of autophagy-related genes have also been reported to be plausible mechanisms of NP-mediated autophagic response^30^,^35^,^36^,^37^. For example, NPs of different chemical composition, such as metal oxides^30^,^36^,^37^, graphene quantum dots^38^, and fullerenes^30^,^39^, could evoke autophagy in a photo-activated- and ROS-dependent manner. Regardless of the mechanism(s) of action, autophagy activity of NPs, alone or in combination with chemotherapeutic drugs, promises to improve cancer therapeutic strategies^34^,^35^,^36^,^37^. Patients would greatly benefit from the development of new strategies for the controlled induction of autophagy in cancer cells^35^,^37^.

While nanomaterials are promising candidates for cancer therapy, their molecular mechanisms of action and the optimal conditions for controlling defined cellular outcomes by NPs are unclear^33^,^34^,^35^,^36^,^37^. Recently, we and others, reported that N-TiO_2_ NPs exhibit remarkable ROS-dependent cytotoxic and apoptotic activities upon visible-light irradiation in several cancerous cell lines, including HeLa and K562 cells^18^,^19^,^40^. In this study, we optimized this NP-based PDT system using visible-light as a safe, remote and controllable stimulator of well-dispersed N-TiO_2_ NPs to trigger autophagy or other cell responses in K592 human leukemia cells and human peripheral lymphocytes^28^,^31^,^35^. We chose the human leukemia cell line K562 because this experimental model of chronic myelogenous leukemia (CML) enables simultaneous evaluation of multiple cellular outcomes. This includes the differentiation toward different lineages (i.e., erythroid, macrophage and megakaryocyte lineages) and various death modes (i.e., apoptosis, autophagy, necrosis and necroptosis)^41^. Our results reveal that upon visible-light irradiation of N-TiO_2_ NPs, an ROS-mediated autophagic response occurred that could be fine-tuned to selectively induce differentiation or apoptosis in leukemia K562 cells.

## Results

### Characterization of N-TiO_2_ NPs and cell culture treatment

**Figure 1:**
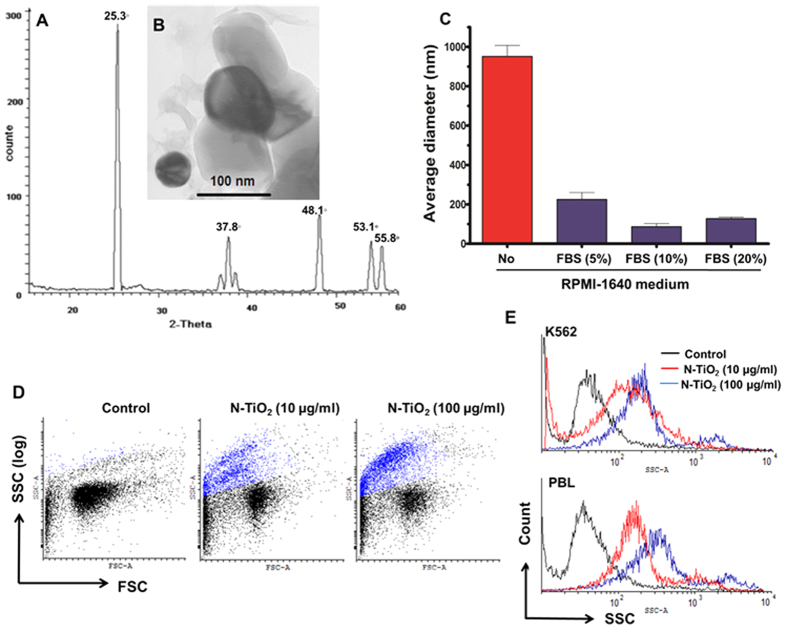
Physicochemical characterization of N-TiO_2_ NPs and their cellular uptake into K562 cells and PBLs. XRD peak (**A**) and TEM image (**B**) of N-TiO_2_ NPs powder. (**C**) Characterization of N-TiO_2_ NPs (1 mg/ml) in aqueous solutions. The average hydrodynamic sizes of NP in cell culture medium (with or without FBS) were measured using DLS, as described in the Materials and Methods. In these experiments, N-TiO_2_ NPs were dispersed in RPMI-1640 medium and sonicated for 10 min, then vortexed and different concentrations of FBS (0–20%, v/v) were added. Hydrodynamic sizes were analyzed by DLS. (**D**,**E**) Analyses of N-TiO_2_ NP incorporation into the cytoplasm of K562 cells by flow cytometric light scatter. The cells were treated with 10 and 100 μg/ml N-TiO_2_ under dark conditions for 3 h, and then the fluorescent intensity of both SSC (**D**,**E**) and FSC (**D**,**E**) was changed and analyzed using flow cytometry in K562 cells and PBLs.

The physicochemical properties of the synthetized N-TiO_2_ NPs have been reported in our previous study^17^ and showed XRD peaks at 2θ = 25.3°, 37.8°, 48.1°, 53.1°, and 55.8° (Fig. 1A), which corresponded to the anatase form of TiO_2_. The average sizes of N-TiO_2_ NPs were estimated between 60–80 nm by TEM analysis (Fig. 1B). Dispersing N-TiO_2_ NPs in deionized water (dH_2_O) resulted in the formation of aggregates/agglomerates as observed using scanning electron microscopy (SEM; Fig. S1A) and DLS (Fig. S1B). Preparations of N-TiO_2_ NPs in cell culture medium without any dispersing agents led to slightly higher hydrodynamic size (951 ± 55 nm) than when dispersed in dH_2_O (773 ± 24 nm; Fig. 1C). These nearly-micron-size aggregates/agglomerates are not recommended for investigating biological effects of NPs because of their cytotoxicity and complex cell delivery pathways^42^,^43^. Modifications to the surface of NPs or protein adsorption usually result in better dispersion of NPs and consequently prevent these nonspecific cytotoxic effects^31^,^42^,^44^. Upon sonication of the NP dispersion, the addition of 10% FBS minimized the aggregation of N-TiO_2_ NPs in culture medium (Fig. 1C)^42^,^45^. When compared to RPMI-1640, the N-TiO_2_ NPs in the medium stabilized with 10% FBS had, on average, a 10-fold lower hydrodynamic size distribution (92–98 nm), which is suitable for cellular penetration based on previous research^14^,^15^,^16^. Consistent with these results, the measured zeta potential values for well-dispersed N-TiO_2_ NPs showed more negative charges, which suggest NP disaggregation (Table 1).

The cytoplasmic uptake of well-dispersed NPs by both K562 leukemia cells and isolated peripheral blood lymphocytes was confirmed by flow cytometry and indicated similar uptake of NPs by cancer and normal cells (Fig. 1D,E). A detectable increase in the mean fluorescent intensity (MFI) of SSC and a decrease in FSC (Fig. 1D) were observed after K562 cells were exposed to N-TiO_2_ NPs for 3 h. This is believed to be a result of light reflection that resulted from cellular NP uptake^46^. Cellular uptake of well-dispersed N-TiO_2_ was enhanced further by increasing the NP concentration from 10 to 100 μg/ml in K562 cells (Fig. 1E and Table 1). The fold increase in SSC of K562 cells exposed to well-dispersed N-TiO_2_ was also higher than aggregated N-TiO_2_. Thus, dispersed N-TiO_2_ NPs are more effective in penetrating cells (Table 1). Similar results were obtained when normal PBLs were exposed to different concentrations of N-TiO_2_ NPs (Fig. 1E). The SSC MFI ratio, which represents cellular uptake of 10 and 100 μg/ml N-TiO_2_ NPs into PBLs, was 5.8- and 7.3-fold higher than control, respectively (Fig. 1E). Our data revealed that both the degree of NP dispersion and NP concentration affected cellular uptake of N-TiO_2_ NPs, providing two parameters that can affect NP cellular content and biological responsiveness.

### Photo-activated N-TiO_2_ NPs augment ROS levels and inhibit growth in a light energy- and NP concentration-dependent manner

**Figure 2:**
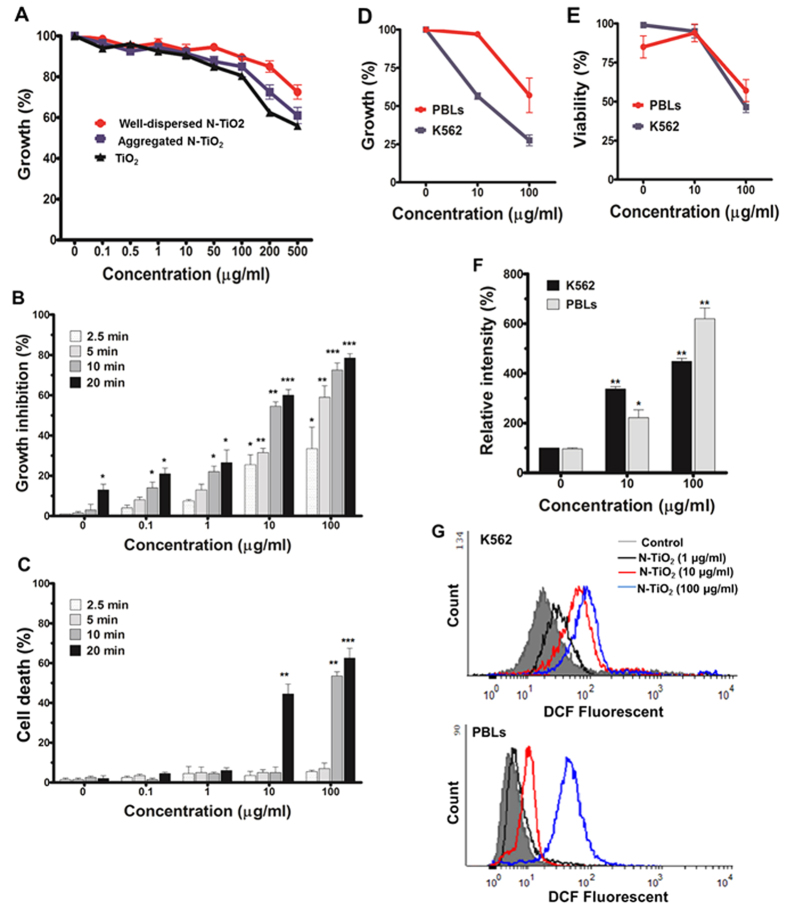
Anti-cancer and ROS producing effects of various concentrations of N-TiO_2_ and TiO_2_ NPs alone or in combination with different visible light energy. (**A**) The cells were exposed to different concentrations of aggregated and well-dispersed forms of NPs (0.1–500 μg/ml) for 24 h, then the growth inhibition was estimated using the trypan blue exclusion test. All the results are from three independent experiments ± SD, each performed in duplicate. (**B**,**C**) The K562 cells were treated with different concentrations of N-TiO_2_ NPs (0–100 μg/ml) for 3 h, then illuminated with various doses of visible light (for 2.5–20 min) using a 55W Xenon lamp. Growth inhibition (**B**) and cell death (**C**) were estimated at 24 h after irradiation. All the results are from four independent experiments ± SD, each performed in triplicate. Statistical analyses of data for each sample are relative to the corresponding control cells (cells that received irradiation without NP). *P < 0.05, **P < 0.01 and ***P < 0.001. (**D**,**E**) Effects of different doses of N-TiO_2_-based PDT in normal and leukemia cells. Three hours after exposing K562 cells and normal PBLs to N-TiO_2_ (10 and 100 μg/ml), they were subjected to 10 min of irradiation (12 J/cm^2^), and growth (**D**) and viability (**E**) were determined at 24 h after PDT. The results are the means of three independent experiments ± SD and the results are presented as the percent of control cells (cells that received irradiation without NP). For ROS analysis, the PDT-treated cells were collected at 30 min after irradiation and stained with DCFH-DA, and DCF fluorescence intensities were then analyzed on a plate reader fluorimeter (**F**). The results are expressed as the fold increase (%) in fluorescence intensity (arbitrary unit, a.u.) of control cells. Error bars are the mean ± SD with *P < 0.001 and **P < 0.0001 *versus* control cells. ROS levels of both K562 and PBL cells were also analyzed using flow cytometry (**G**) at 30 min after illumination (12 J/cm^2^) of N-TiO_2_ (1–100 μg/ml). The peaks from each experiment were for better comparing the changes in MFI of DCF. The results of PBLs are from one representative healthy sample.

We have designed a PDT system composed of N-TiO_2_ NPs as photosensitizer and visible light as a remote stimulator to fine-tune cellular outcomes. We chose the human leukemia cell line K562 because this experimental model of CML allows the simultaneous evaluation of cell differentiation and/or cell death. We studied the cytotoxicity of well-dispersed versus aggregated forms of N-TiO_2_ and non-doped TiO_2_ in K562 cells (Fig. 2A). Well-dispersed N-TiO_2_ had no growth inhibitory effect up to concentrations of 100 μg/ml in the absence of visible light (Fig. 2A). However, the combination of well-dispersed N-TiO_2_ NPs with visible light resulted in a marked reduction in growth of K562 cells (Fig. 2B), which was incremental with enhancing concentrations of N-TiO_2_ and increasing light doses (3–24 J/cm^2^). Irradiation of well-dispersed forms of N-TiO_2_ (10 μg/ml) for 2.5, 5, 10, and 20 min resulted in a 25 ± 4%, 30 ± 3%, 56 ± 2%, and 60 ± 3% reduction in growth, respectively, compared to control cells as determined after 24 h (Fig. 2B). Visible light alone (Fig. 2B) failed to have a growth inhibitory effect on the cells at up to 10 min of illumination (3–12 J/cm^2^) and a marginal (15 ± 3%) reduction in cell numbers was observed after 20 min of visible light irradiation (24 J/cm^2^). The viability of K562 cells remained unaltered at low concentrations of NP and short irradiation times. However, a substantial decrease in cell viability was observed at doses of 10 μg/ml (24 J/cm^2^ irradiation) and 100 μg/ml (12 and 24 J/cm^2^ irradiation), suggesting that only high PDT doses were able to initiate cell death responses in K562 cells (Fig. 2C). Thus, these results suggest that induction of cell death could be regulated by both the applied concentration of well-dispersed N-TiO_2_ NPs and light exposure duration and intensity. Also, anti-cancer effects of well-dispersed N-TiO_2_ NPs is directly dependent on light irradiation (Fig. S2).

To test the selectivity of such treatment for cancer cells, we compared the effect NP concentrations (10 and 100 μg/ml) and PDT (12 J/cm^2^ or 10 min irradiation) had on K562 with that of human PBLs (Fig. 2D,E). While growth of K562 cells was profoundly inhibited at low NP doses (10 μg/ml), this was not observed in the PBL cells (Fig. 2D). A 48 ± 15% reduction in PBL numbers was observed at high NP concentrations (100 μg/ml), but this was less than the 73 ± 5% reduction seen with K562 cells. Similar to our previous results (Fig. 2C), low PDT failed to significantly affect viability of K562 while an unexpected increase in viability was observed in PBL (Fig. 2E). At high PDT doses (100 μg/ml, 12 J/cm^2^), viability decreased in both PBL (45 ± 10%) and K562 cells (52 ± 3%). Thus, selective growth inhibition of K562 leukemia cells was achieved at low dose PDT (10 μg/ml, 12 J/cm^2^) without any adverse effects on normal PBLs.

The induction of oxidative stress via direct generation of ROS is a major PDT outcome and also a cause of PDT cytotoxicity^6^,^8^,^27^. To evaluate the effects of N-TiO_2_-based PDT on ROS production, we measured the intracellular levels of peroxides using DCFH-DA staining of the cells using fluorimetry (Fig. 2F) and flow cytometry (Fig. 2G). Similar to growth inhibition, a concentration-dependent increase in fluorescence DCF intensity was recorded following exposure of K562 and PBL cells to well-dispersed N-TiO_2_ (Fig. 2F). Flow cytometric analysis for DCF intensity (ROS level) provided a better view on differential effects of N-TiO_2_-based PDT in PBL and K562 cells (Fig. 2G). While minimal fluorescence signals were registered in PBL from all three healthy volunteers, K562 cells recorded high fluorescence background indicating that endogenous basal levels of ROS were significantly higher in K562 cells than in PBLs. In both cells, DCF intensity increased along with increasing PDT dose levels, but the ROS kinetics and augmentation levels were different in PBL and K562 (Fig. 2G). Higher endogenous ROS levels may explain the increased sensitivity of K562 leukemia cells at lower N-TiO_2_ NP concentrations. PDT-induced ROS levels surpass a critical threshold level that triggers K562 cell death, whereas PBLs with low endogenous ROS levels are more tolerant.

### Different concentrations of photo-activated NPs induce distinct cellular fates

**Figure 3:**
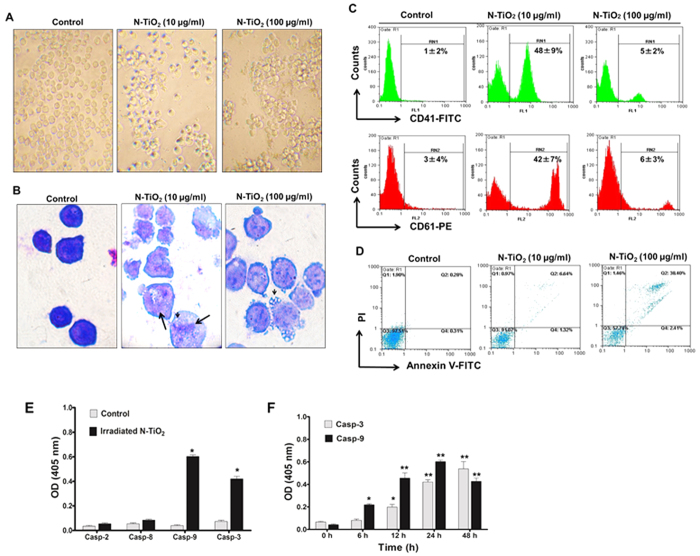
Effects of different doses of N-TiO_2_-based PDT on differentiation and apoptosis in K562 cells. K562 cells were exposed to low (10 μg/ml, 12 J/cm^2^) and high (100 μg/ml, 12 J/cm^2^) doses of PDT for 24 h, then morphological changes with (**B**) or without (**A**) Wright-Giemsa staining were studied using light microscopy (magnification 60× and 40×, respectively). Arrows indicate polynucleation and giant cells, and arrow heads refers to cytoplasmic vacuolization. (**C**) The expression of CD41 and CD61 markers were measured after 24-h exposure to PDT with 10 and 100 μg/ml concentrations of NP using flow cytometry with the corresponding antibodies against each antigen. The results are presented as the mean ± SD of three independent experiments. (**D**) At 24 h after PDT by N-TiO_2_ (10 and 100 μg/ml), the cells were harvested and Annexin-V/PI double staining was used to detect apoptosis via flow cytometry. Caspase activity (**E**,**F**) was also calculated by measuring the absorbance at 405 nm (OD). Following high PDT of K562 cells for 24 h, the cytosolic extracts were collected, incubated with respective caspase substrates, then the activities of caspase-2, −3, −8 and −9 were determined, as described in the Materials and Methods. The data are presented as the OD of cells exposed to high PDT *versus* control unexposed cells. Only the caspase-9 and -3 activity differed significantly between control and PDT-exposed cells (*P < 0.0001). (**F**) Time-dependent activities of caspase-9 and −3 were also measured following exposure of K562 cells to high PDT. The activity of caspase-9 and −3 differed significantly after 6 h (*P < 0.001) and 12 h (**P < 0.0001), respectively, from control cells (0 h).

We explored the molecular mechanisms activated by PDT with different concentrations of N-TiO_2_ that resulted in the induction of distinct cellular outcomes. Morphological changes induced by different NP concentrations (10 and 100 μg/ml) were distinct in K562 cells (Fig. 3) and PBLs (Fig. S3). Following exposure of K562 cells to 10 μg/ml concentrations of photo-activated N-TiO_2_, some cells acquired significant changes in cell size and cell-to-cell adherence (Fig. 3A), suggesting the occurrence of cellular differentiation. Giemsa staining showed morphological features of megakaryocytes, including large cells with poly-lobulated nuclei, cytoplasm vacuolization and increased nuclear-to-cytoplasm ratio (Fig. 3B). Flow cytometric analyses of PDT-treated cells identified high expression of CD41 (αIIb chain, platelet GPIIb) and CD61 (β3 integrin, platelet GPIIIa), which are typical markers for megakaryocytes and platelets. These changes were found in K562 cells that were exposed to only 10 μg/ml doses of photo-activated N-TiO_2_ (Fig. 3C). Differentiation toward the erythroid linage was excluded by benzidine staining (Fig. S3).

Increased cell debris was observed in K562 cells (Fig. 3A) and PBLs (Fig. S3) exposed to 100 μg/ml photo-activated N-TiO_2_. Flow cytometric analysis of Annexin-V/PI failed to detect signs of cell death following low PDT (10 μg/ml N-TiO_2_). However, 100 μg/ml photo-activated N-TiO_2_ was a potent inducer of apoptotic cell death in both K562 cells (41 ± 8%, Fig. 3D) and PBLs (40 ± 11%, Fig. S3). We measured the activation of caspases involved in death receptor (caspase-2, −8) and mitochondrial (caspase-9) apoptosis pathways. Exposure of K562 to high concentrations of photo-activated N-TiO_2_ (100 μg/ml) resulted in a significant (P < 0.001) activation of caspase-9 (15.3-fold of control) and −3 (6.7-fold of control), but not caspase-2 and −8 (Fig. 3E). Activation of caspase-9 was first detected at 6 h, it peaked at 24 h, and then activation decreased at 48 h (Fig. 3F). A significant increase in caspase-3 started at 12 h and increased exponentially up to 48 h. This pattern of caspase activation confirmed the involvement of the mitochondrial pathway of apoptosis, where caspase-9 activation results in a marked activation of caspase-3^10^,^47^.

### Autophagy is activated following photo-activation of N-TiO_2_ by visible light

**Figure 4:**
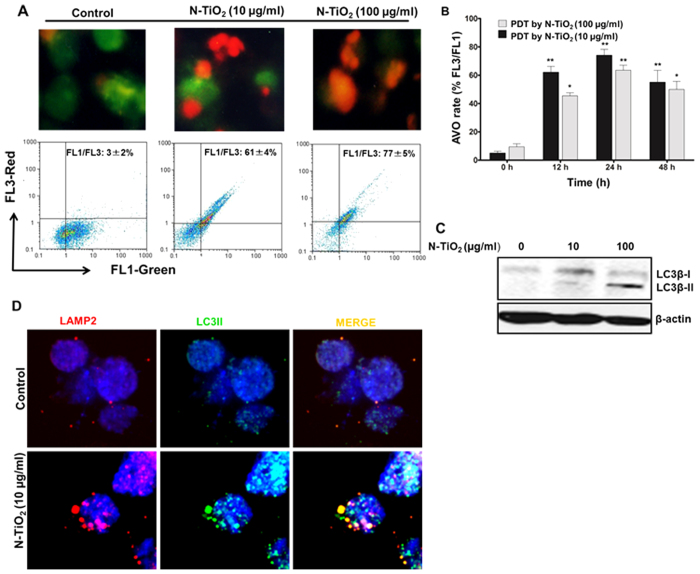
Autophagic effects of light-irradiated N-TiO_2_ in K562 cells. (**A**) Cells were exposed to N-TiO_2_ (10 and 100 μg/ml) and irradiated for 10 min. They were then stained with acridine orange (AO) and formation of AVO was detected using light microscopy after 24 h. Flow cytometry was also used for quantification of AVO formation at the same time (**A**,**B**). The results were presented as the percentage of FL3/FL1 intensity (mean ± SD). Statistical significance were expressed as *P < 0.0001 **P < 0.0001 compared with corresponding control cells (0 h). (**C**) The protein expression level of LC3B, a typical marker of autophagy, was monitored at 24 h after exposing K562 cells to photo-activated N-TiO_2_ (10 and 100 μg/ml) using specific monoclonal antibody by immunoblotting. The actin was used as loading control. Data are representative of one typical experiment. (**D**) Immunofluorescence staining for LC3-β and LAMP2 (**D**) in K562 cells exposed to photo-activated N-TiO_2_ (10 μg/ml) for 24 h. The LC3-β (green) and LAMP2 (red) punctuations and colocalization of LAMP2 with cytosolic LC3 (orange) were detected under a confocal microscope, as described in the Materials and Methods. Nuclei were stained by DAPI (blue).

**Figure 5:**
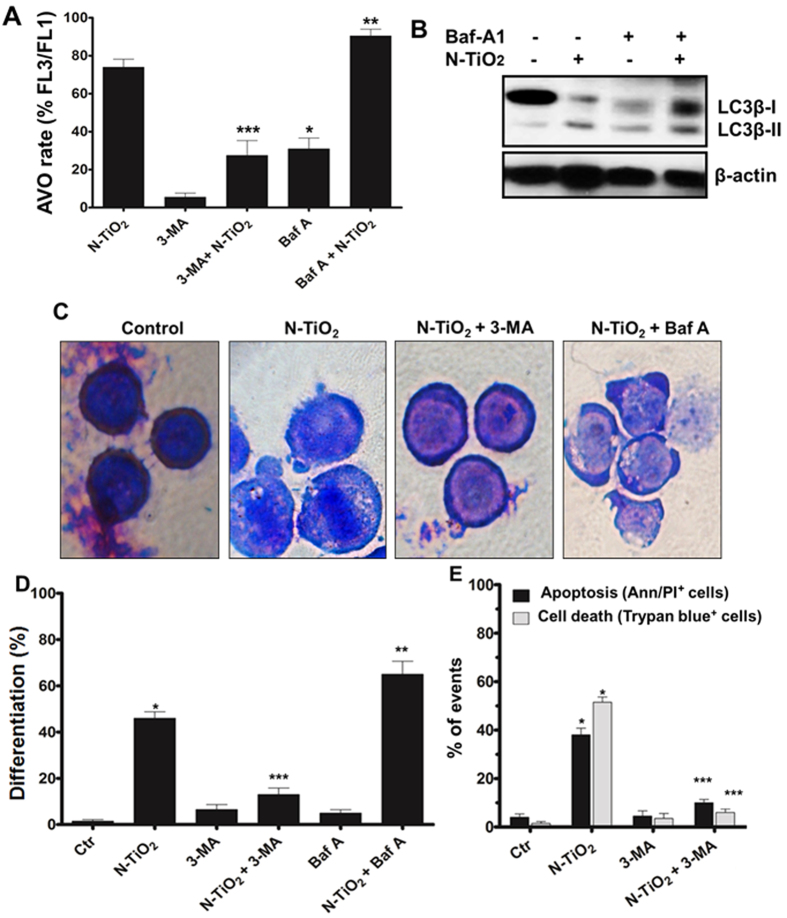
Effects of autophagy inhibitors on differentiation and cell death induced by N-TiO_2_-based PDT in K562 cells. (**A**,**B**) The cells were exposed to high PDT (100 μg/ml N-TiO_2_, 12 J/cm^2^) in the presence or absence autophagy inhibitors (3-MA, 1 mM or Baf A, 100 nM) for 24 h, and formation of AVO and LC3-β fragmentation were studied using flow cytometry (A) and immunoblotting (the immunoblot is the crop of the band and representative of three experiments which have been run on the same conditions) (**B**), respectively. (**C**,**D**) To study the effects of autophagy inhibition on differentiation, the cells exposed to PDT with 10 μg/ml N-TiO_2_ (12 J/cm^2^), and/or autophagy inhibitors (3-MA, or Baf A) for 24 h, then differentiation was evaluated using Wright-Giemsa staining (**C**) and the percentage of CD41^+^ cells (**D**) was calculated using flow cytometry. (**E**) K562 cells exposed to high PDT (100 μg/ml N-TiO_2_, 12 J/cm^2^), in the presence or absence of autophagy inhibitor (3-MA, 1 mM) for 24 h, and apoptosis and cell death were determined using flow cytometry analysis of AnnexinV/PI^-^positive cells and counting trypan blue-positive cells, respectively. Significant combinations were expressed as *P<0.001 (compared with control untreated cells) and **P < 0.05 and ***P < 0.01 (compared with cells exposed to photo-activated N-TiO_2_). All data in this Figure are from three independent experiments ± SD, each performed in triplicate.

Recent evidence suggests that ROS generated by PDT could result in autophagy induction^9^,^27^,^28^. In our model system, the occurrence of autophagy was first visualized by the formation of AVOs using acridine orange as a fluorescent dye that is specific for acidic compartments^48^,^49^. Fluorescence microscopy provided morphological confirmation of AVO formation in response to both low and high concentrations of photo-activated N-TiO_2_ (Fig. 4A) and flow cytometric analysis detected increased red fluorescence between 12–48 h of PDT (Fig. 4A,B). Vigorous PDT induced increased amounts of AOV formation, suggesting that the level of autophagy induction could be fine-tuned, depending on the applied PDT dose (Fig. 4B). Microtubule-associated protein light chain 3 (LC3) is a reliable autophagy marker and was analyzed by Western blotting (Fig. 4C). Upon induction of autophagy, cytosolic LC3 (LC3-I, top band) is cleaved by Atg4 and subsequently conjugated to phosphatidylethanolamine (LC3-II, bottom band) by Atg3, which allows LC3-II to insert into the membrane of autophagosomes^22^,^23^. LC3-II levels were observed after a 24-h exposure to both low and high PDT (Fig. 4C). Punctate signals of LC3-II were observed in both the cytosol and nucleoplasm of K562 cells exposed to PDT (Fig. 4D). The occurrence of autophagy flux was assessed by the detection of lysosomal marker LAMP2 (lysosome-associated membrane protein 2) (Fig. 4D). We have observed that punctuate LAMP2 co-localizes with cytosolic LC3-II (Fig. 4D). The autophagy flux was further confirmed using the lysosomal proton pump inhibitor bafilomycin A1 (Baf A). Autophagy flux was confirmed by the increased formation of LC3-II following co-treatment with photo-activated N-TiO_2_ and Baf A1 (Fig. 5A,B). Additionally, both 10 and 100 μg/ml concentrations of photo-activated N-TiO_2_ induced autophagy in PBLs (Fig. S5). However, the level of autophagy induction following exposure of PBLs to NPs was less than that in K562 cells (Fig. S5A), and the autophagic effects of 10 μg/ml photo-activated N-TiO_2_ in PBLs was not associated with growth inhibition (Fig. 2D) or morphological changes (Fig. S3).

### Induction of autophagy is required for the differentiation and apoptosis induced by photo-activated N-TiO_2_

To address the importance of early autophagy activation, we used the autophagy inhibitor 3-methyladenine (3-MA), a phosphatidylinositol 3-kinase inhibitor that blocks autophagy at the early stages by sequestering cargo, or Baf A1 that suppresses autophagy at later stages by preventing the phagosome formation. Autophagy inhibition by 3-MA prevented AVO formation (Fig. 5A) and LC3-II accumulation in K562 cells exposed to photo-activated N-TiO_2_ (Fig. S6). Wright-Giemsa staining indicated that 3-MA blocked the morphological changes resembling megakaryocytic differentiation (Fig. 5C), and this coincided with a noticeable decrease in the percentage of K562 CD41^+^ cells (P < 0.01) that were treated with a combination of 3-MA and low PDT (Fig. 5D). While Baf A1 (100 nM) blocked late-stage autophagic flux (Fig. 5A,B), it significantly (P < 0.05) increased the differentiating effects of photo-activated N-TiO_2_, as denoted by morphology (Fig. 5C) and the percentage of CD41^+^ cells (Fig. 5D).

To clarify the cross-talk between autophagy and apoptosis, 3-MA was used in co-treatment with N-TiO_2_ (100 μg/ml). Notably, 3-MA blocked the number of dead cells (trypan blue-positive cells) induced by photo-activated N-TiO_2_ (100 μg/ml) and increased viability, suggesting that autophagy acts as a death pathway at 100 μg/ml of photo-activated N-TiO_2_ (Fig. 5E). Apoptosis was also diminished by 3-MA, which suggests an upstream role of autophagy in the induction of apoptosis. The results suggest an essential requirement for early-stagy autophagy in determining the cellular outcomes induced by N-TiO_2_-based PDT.

### ROS is upstream of autophagy, terminal differentiation and apoptosis in NP-based PDT

**Figure 6:**
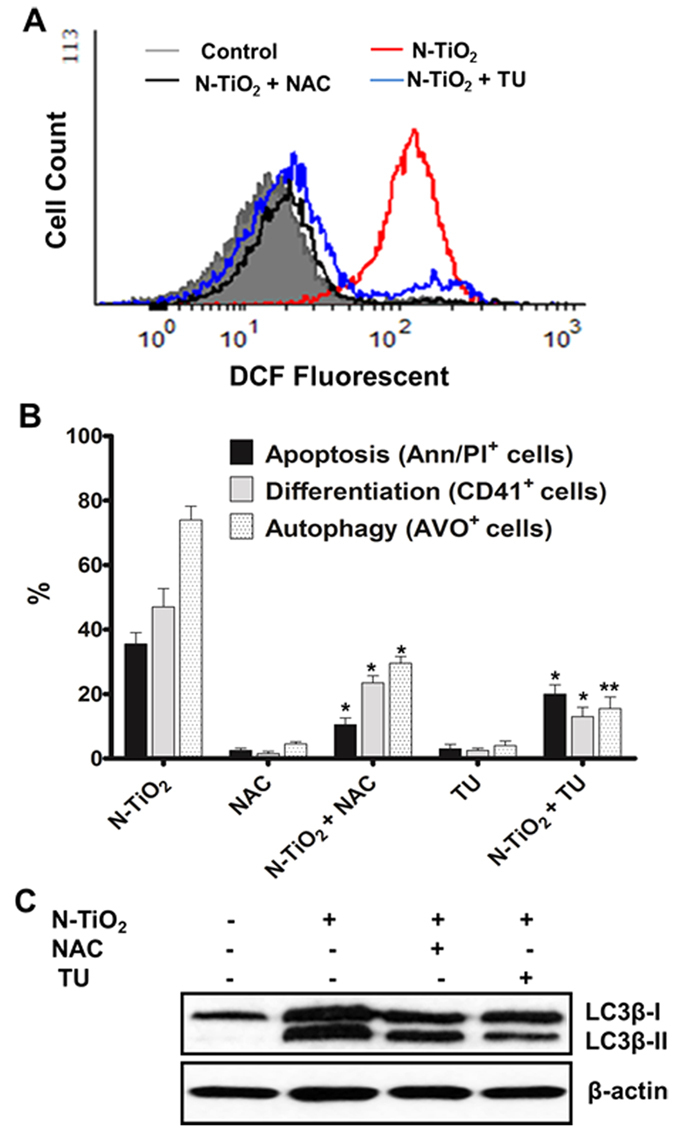
Inhibitory effect of ROS scavengers on cell fate induced by N-TiO_2_-based PDT. (**A**) K562 cells were preincubated with NAC (10 mM) or thiourea (15 mM) for 30 min, and/or exposed to high PDT (100 μg/ml N-TiO_2_, 12 J/cm^2^). Thirty minutes later, ROS levels were determined by staining with DCFH-DA and analyzing DCF fluorescence intensities using flow cytometry. (**B**,**C**) Effects of ROS scavengers on different cellular outcomes were examined following preincubation of the cells with NAC (10 mM) or thiourea (15 mM) for 30 min and then exposing to N-TiO_2_-based PDT for 24 h. To analyze apoptosis, cells exposed to high PDT (100 μg/ml N-TiO_2_, 12 J/cm^2^) and the percentage of Annexin V/PI-positive cells were determined using flow cytometry. For differentiation, the cells were exposed to photo-activated N-TiO_2_ (10 μg/ml) and the percentage of CD41^+^ cells was evaluated using flow cytometry (**B**). For autophagy, the cells exposed to photo-activated N-TiO_2_ (10 μg/ml) and AVO formation (**B**) and up-regulation of LC3 II (**C**) were assessed using flow cytometry and Western blotting ((the immunoblot is the crop of the band and representative of three experiments which have been run on the same conditions), respectively. All data in this Figure are from three independent experiment ± SD each performed in duplicate. *P < 0.01 and **P < 0.001 compared with cells exposed to photo-activated N-TiO_2_.

To elucidate a link between oxidative stress and the observed cellular outcomes induced by different PDT doses, two ROS scavengers, N-acetyl-L-cysteine (NAC) as a potent inhibitor of H_2_O_2_ and thiourea (TU), as an effective scavenger of OH· and H_2_O_2_, were used. Pretreatment for 30 min with either scavenger inhibited ROS augmentation (Fig. 6A). This was accompanied by a substantial reduction in annexin V/PI^+^ apoptotic cells (Fig. 6B). Similar results were obtained after preincubation of PDT exposed cells with TU (Fig. 6B). Moreover, both inhibitors alleviated the differentiation (CD41^+^ cells) and autophagy (LC3 II lipidation and AVO formation) in K562 cells (Fig. 6B,C), confirming that ROS is the upstream driver of these cellular events. TU, which was less potent than NAC in reducing apoptosis (Fig. 6A), was able to prevent autophagy and differentiation more efficiently than NAC. The mechanism by which PDT by N-TiO_2_ generates ROS and the impact of different ROS species on cell fate warrants further investigation.

## Discussion

Although the intrinsic cytotoxicity of semiconductor metal-oxide NPs, such as TiO_2_, alone^32^,^33^,^34^ or in combination with PDT^19^,^20^,^32^, has been exploited in anti-cancer therapies, lack of selectivity and uncontrollable cellular outcomes remains a challenge. In this study, we showed a time- and cost-effective PDT^17^,^18^,^19^,^20^ with well-dispersed N-TiO_2_ NPs that elicited a controlled autophagy response and ROS generation. Unlike most metal oxide, NPs and TiO_2_ dopants, which are cytotoxic in the absence of light, N-doped TiO_2_ NPs are biologically inert and non-toxic in the absence of light^18^,^19^,^20^,^40^, and they generate ROS upon visible light irradiation^18^,^19^,^20^,^21^. Aggregation of metal oxide NPs (e.g., TiO_2_) can profoundly affect their cellular uptake and biological behavior (e.g., autophagy effects) in a hydrodynamic size- and dispersity-dependent manner^28^,^31^,^42^,^44^,^50^. Protein adsorption onto the surfaces of NPs promoted the formation of non-toxic well-dispersed N-TiO_2_ NPs^31^,^42^,^43^,^44^,^51^, and despite conflicting reports^42^,^52^,^53^, increased cellular uptake in our cell models. The proteins of FBS, such as albumin, can adsorb onto surface of NPs and prevent aggregation in a concentration-dependent manner^43^ (Fig. 1C). However, it should be note that FBS contains many other supplements such as amino acids, lipids and ions that may interfere with its anti-aggregating potential in some concentrations^44^,^50^,^54^ like the results we observed by adding 20% FBS (Fig. 1C).

The cytotoxic effects of metal oxide NPs are mostly mediated by ROS production through their surfaces ability to catalyze oxidation reactions or through damage to cellular organs^4^,^9^,^18^,^28^,^29^,^55^. Similar to recent studies^18^,^19^, we used low concentrations of well-dispersed N-TiO_2_ NPs (1–100 μg/ml), which became cytotoxic exclusively upon exposure to visible light (Fig. 2). All effects of NPs were blocked by ROS scavengers (Fig. 6), strongly suggesting that oxidative stress and ROS formation are the main trigger for the cellular changes induced by photo-activated N-TiO_2_. PDT-mediated ROS strategies have been used in cancer therapy^6^,^7^,^8^,^27^, with the type and quantity of ROS members produced by PDT being dependent on the light dose and concentrations of the photosensitizer used^7^,^8^,^27^. In addition, PDT conditions have been shown to have an impact on changing cell fates and osteoblast differentiation^7^. We showed that at constant light energy (12 J/cm^2^), well-dispersed N-TiO_2_ NPs generated a cellular ROS response in a concentration-dependent manner, which resulted in distinct cellular outcomes in K562 and PBL cells (Figs 2 and 3). While robust ROS increases above a certain threshold triggered cell death, more subtle cellular responses were induced at low NP doses (10 μg/ml; Fig. 2D,E). This was not the result of different N-TiO_2_ NP cellular content in both cell types (Fig. 1E), but it may be associated with higher endogenous ROS levels observed in K562 leukemia cells (Fig. 2G). Contrary to normal cells, tumor cells frequently experience chronic oxidative stress and ROS challenges that render them vulnerable to toxic thresholds, while normal cells can maintain redox homeostasis and survive^56^,^57^,^58^. Other contributing factors for the selective toxicity of TiO_2_ NPs may include differences in mitochondrial activity^35^,^36^,^39^ and lysosomal capacity^59^ between normal and malignant cells.

Autophagy is a desired outcome and a main mechanism involved in NP effects^21^,^35^,^36^,^37^,^39^. We identified early induction of autophagy and functional autophagy flux by photo-activated N-TiO_2_ in K562. Depending on the type of stress and the cellular micro-environment, autophagy could protect or destroy cells^21^,^22^,^23^,^52^,^60^,^61^. In our cell models, these opposing autophagy responses were dependent on the concentration of photo-activated N-TiO_2_ NPs, with low (10 μg/ml) and high (100 μg/ml) concentrations of N-TiO_2_ inducing autophagy-dependent differentiation or autophagy-associated apoptosis, respectively (Figs 3 and 4). Different ways have been proposed to trigger an autophagic response via NPs^33^,^34^,^35^,^62^. Our well-dispersed N-TiO_2_ NPs did not show intrinsic potential to induce autophagy at concentrations below 100 μg/ml (unpublished data), but they were able to induce autophagy by photo-activation stimulated ROS generation (Fig. 6). ROS damage to different macromolecules and organelles can lead to different cellular responses^28^,^32^,^33^,^34^,^35^. Iron oxide and gold-coated iron NPs were reported to elicit high levels of ROS, resulting in mitochondrial dysfunction, autophagy, and apoptosis^30^,^34^,^35^. Similarly, TiO_2_ NP-generated ROS induces ER stress and mitochondrial damage, which results in autophagic cell death^9^. PTD using TiO_2_ NPs was shown to trigger both extrinsic and intrinsic apoptotic pathways in different cell lines^5^,^59^,^63^. In our study, autophagy induced by photo-activated N-TiO_2_ involved the mitochondrial-dependent apoptosis pathway (Fig. 4). Autophagy induction may directly evoke apoptosis by facilitating activation of caspases or interaction with mediators of apoptotic pathways, such as the BCL2 protein family^64^. Moreover, autophagy may indirectly influence apoptotic responses via autophagy-dependent degradation of cytotoxic and aggregated proteins^64^.

The importance of nuclear LC3-II puncta observed in our study is not clear, but may involve nuclear-cytoplasmic shuttling of LC3 under cellular stress, which was recently reported^65^,^66^. In fact, the microtubule-associated protein LC3 has pleiotropic functions by interacting with the guanine nucleotide exchange factor SOS1 and the Ca2^+^-sensing protein caldendrin, and by regulating fibronectin mRNA levels^65^,^66^. In addition, a direct interaction of bioactive silica NPs with LC3-II has been recently reported that is associated with autophagy stimulation and osteoblast differentiation^67^. Future studies will focus on the biological relevance of nuclear accumulation and LC3 shuttling on the cytotoxicity of photo-activated N-TiO_2_ NPs.

Autophagy has an essential role in cell differentiation^68^,^69^,^70^,^71^,^72^. The genetic knockout of autophagy-related (*ATG*) genes revealed pivotal functions of autophagy during cell differentiation and development^72^. For example, autophagy plays an upstream role during adipocyte, monocyte, erythroid and neuronal differentiation^23^,^68^,^69^,^70^,^71^,^72^,^73^, and it was recently shown to induce megakaryocyte differentiation^69^,^71^. Autophagy-associated self-digestive processes may aid morphological changes related to differentiation (disappearance of mitochondria and/or nuclei) and cell survival during differentiation^68^,^70^,^71^. Here, we report for the first time the ability of photo-activated N-TiO_2_ NPs to induce terminal megakaryocyte differentiation in K562 leukemia cells. Upon exposure to low concentrations of photo-activated N-TiO_2_ NPs, autophagy was essential for megakaryocytic differentiation in K562, and this effect was blocked by the autophagy inhibitor 3-MA (Fig. 5). However, Baf A1 sequesters the late stages of autophagy and enhances megakaryocyte differentiation induced by NP-based PDT (Fig. 5). This may be a result of the accumulation of molecular mediators promoting differentiation, as reported for tetrandrine-induce differentiation^73^, or it could result from Baf A1-specific effects independent of autophagy^74^. Autophagy pathways can alter signaling pathways affecting the differentiation state of leukemia cells^73^ and pharmacological strategies target autophagy activation to promote terminal differentiation and cell death of leukemic cells^73^,^75^,^76^,^77^. Our discovery of the autophagy-dependent differentiating potential of photo-activated N-TiO_2_ may have therapeutic advantages for certain types of leukemia^76^,^78^.

## Conclusion

**Figure 7:**
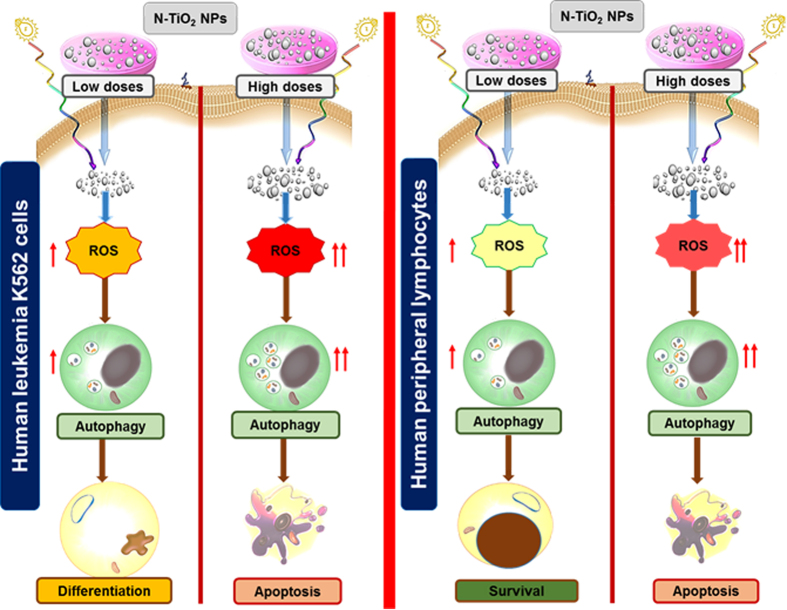
Schematic overview of the cellular outcomes induced by N-TiO_2_-based PDT in human leukemia K562 cells and PBLs. At a constant light energy (12 J/cm^2^), 10 μg/ml (low dose) and 100 μg/ml (high dose) concentrations of well-dispersed N-TiO_2_ NPs lead to different increases in basal (inherent) ROS levels in K562 cells and peripheral lymphocytes (PBLs). Depending on levels of ROS generated by PDT, autophagy induction may be associated with megakaryocytic differentiation or apoptosis in K562 cells. While high doses of PDT (100 μg/ml N-TiO_2_, 12 J/cm^2^) induced autophagy-associated apoptosis in PBLs, low PDT doses did not lead to any growth inhibitory or cytotoxic effects in this human normal-cell model.

We showed the concentration-dependent capability of well-dispersed photo-activated N-TiO_2_ NPs to induce terminal megakaryocyte differentiation or cell death in K562 leukemia cells. These cellular outcomes depend on intracellular ROS levels and are mediated by autophagy (Fig. 7). In this situation, low PDT doses (10 μg/ml N-TiO_2_, 12 J/cm^2^) also increased ROS and autophagy levels in PBLs, but it did not lead to any growth inhibitory or cytotoxic effects in this human normal-cell model. Our combined N-TiO_2_ NPs and PDT strategy permits preferential targeting and controlled photo-activated induction of ROS and autophagy activation in leukemia cells and may represent a novel therapeutic approach for a broad range of other cancer types.

## Materials and Methods

### Reagents

Pure TiO_2_ powder (Cotiox KA-100, anatase: >99%) was supplied by Cosmo Chemical Co. (Incheon, South Korea). Urea was purchased from Merck (Darmstadt, Germany). RPMI-1640 cell culture medium and fetal bovine serum (FBS) were purchased from Biosera (East Sussex, UK). Penicillin and streptomycin were obtained from Cinnagen (Tehran, Iran). Culture flask and plates were obtained from SPL Life Science (South Korea). Phytohemagglutinin (PHA), thiourea (TU), N-acetyl-L-cysteine (NAC), trypan blue, Tris-HCl, propidium iodide (PI), acridine orange (AO), 4′,6-diamidino-2-phenylindole (DAPI), 3-methyladenine (3-MA), bafilomycin A1 (Baf A1), Triton X-100, Tween-20, sodium dodecyl sulfate (SDS), dimethylsulfoxide (DMSO), ethylenediaminetetraacetic acid (EDTA), phenylmethylsulphonyl fluoride (PMSF), 2′,7′- dichlorofluoresceindiacetate (DCFH-DA), and protease inhibitors (aprotinin, pepstatin and leupeptin) were obtained from Sigma (Germany).

### Synthesis and characterization of N-doped TiO_2_ NPs

The N-TiO_2_ NPs were prepared by mechanical mixing of urea with anatase TiO_2_ powder in a 4:1 ratio, as reported previously^17^. Briefly, the materials were annealed under air atmosphere for 1 h at 400 °C with a heating rate of 10 °C per minute and cooled to room temperature (RT), then crushed in an agate mortar to obtain a fine light-yellow powder. The crystal phase composition of N-TiO_2_ NP sample (amounts of the anatase and rutile phases) was determined by X-ray powder diffraction (XRD) using the Siemens X-ray diffractometer D5000 (Siemens AG, Munich, Germany). The size distribution and morphology of sensitized NPs were analyzed by transmission electron microscopy (TEM) (CM 120, Philips, Germany). The hydrodynamic sizes and surface charges of N-TiO2 NPs were determined by dynamic light scattering (DLS) analyzer (Nanotrac Wave model, Microtrac Inc., USA), according to the manufacturer’s instructions. The N-TiO_2_ stock solutions of 1 mg/ml was prepared in deionized water (dH_2_O) or RPMI-1640 medium (with or without FBS) and dilutions of 10–100 μg/ml N-TiO_2_ (in deionized water, RPMI and RPMI + FBS) were used for DLS analysis to prevent multiple scattering.

### Cell culture and PDT condition

The authenticated human erythroid leukemia cell line K562 was obtained from the National Cell Bank of Iran (Pasteur Institute, Tehran, Iran). Cells were cultured in RPMI-1640 medium supplemented with FBS (10%, v/v), streptomycin (100 μg/ml) and penicillin (100 U/ml) at 37 °C in a 5% CO_2_ humidified atmosphere. Normal human peripheral lymphocytes (PBL) from three healthy donors were obtained following gradient centrifugation on Ficoll-Paque PLUS (Sigma, Munich, Germany). Ethics approval was received from the Ethics Committee of the National Institute of Genetic Engineering and Biotechnology (IR.NIGEB.EC.1394.7.27.2)^79^ and “**the methods were carried out in “accordance” with the relevant guidelines (IR.NIGEB.EC.1394.7.27.2) while an informed consent was obtained from all subjects to get PBL”**. The PBLs were cultured in RPMI-1640 medium containing 12.5% FBS, 100 U/ml penicillin and 100 μg/ml streptomycin. PHA (5 μg/ml) was added to induce proliferation of PBL.

For treatment, pure and N-TiO_2_ NP stock suspensions (1 mg/ml) were prepared in RPMI-1640 medium and ultrasonicated for 10 min. Unless otherwise indicated, FBS was used as a stabilizer to prepare well-dispersed NPs^45^. Stock suspensions were freshly diluted to different working concentrations in RPMI-1640 medium containing FBS (10%, v/v). For PDT, cells were exposed to various concentrations of NPs in the dark for 3 h, washed with PBS, and re-suspended in fresh medium containing FBS. Cells were irradiated with a Xenon 55 W lamp and an ultraviolet cutoff filter eliminating UV light λ < 400 nm at 20 mW/cm^2^ resulting in light energies of 3, 6, 12, and 24 J/cm^2^ for 2.5, 5, 10, and 20 min, respectively.

### Treatment condition for inhibitors

For ROS scavengers, TU (15 mM) or NAC (10 mM) were dissolved in RPMI medium and added 30 min before cells were exposed to NPs. For autophagy inhibition, 3-MA (1 mM, dissolved in PBS) or Baf A1 (100 nM dissolved in DMSO) was used in co-treatment with N-TiO_2_. Stock solution of 3-MA (20 mM) was prepared fresh and used after heating to 55 °C to be dissolved completely.

### Cellular uptake of N-TiO_2_

The uptake of N-TiO_2_ NPs was measured by flow cytometry^46^. Side-scattered light (SSC) is usually affected by intracellular structures whereas forward-scattered light (FSC) is normally related to cell size; however, both SSC and FSC also change upon cellular uptake of nanomaterials^46^. The K562 cells were exposed to different concentrations of well-dispersed N-TiO_2_ for 3 h, then cells harvested and analyzed by FACS Calibur™ flow cytometer (BD Biosciences, USA). The SSC and FSC were measured in logarithmic and linear scales, respectively.

### Cell proliferation and cytotoxicity assay

Cell proliferation and cell death were studied by trypan blue exclusion assay. Briefly, the cells were exposed to different concentrations of NPs in the presence or absence of visible-light for different times and then stained with trypan blue (0.4% mg/ml) and counted. Cell numbers and viability were manually calculated after counting the number of alive (unstained) versus dead (blue-stained) cells using a hematocytometer under inverted light microscopy (Olympus, Japan), as reported previously^79^.

### Benzidine staining

The erythroid differentiation of K562 cells was evaluated by benzidine staining of hemoglobin-producing cells. K562 cells were treated, for 24–48 h, with different concentrations of photo-activated N-TiO_2_ or GTP (100 μM) as a positive control. Cell pellets were dissolved in 50 μl benzidine solution (0.2% in 0.5 M acetic acid) containing 3% H_2_O_2_ solution (30%). The mixture was incubated for 20 min and cells were counted to determine the percent of hemoglobin-containing cells^80^.

### Measurement of ROS level

The intracellular levels of ROS were assayed after staining with the fluorescent probe DCFH-DA by both fluorimeter and flow cytometer devices^81^. This colorless probe can easily diffuse into cells and quickly form DCHF by esterase cleavage. The trapped DCHF can be oxidized subsequently with ROS, principally H_2_O_2_, to produce a fluorescent dichlorofluorescein (DCF) molecule. Briefly, cells were exposed to different concentrations of N-TiO_2_ NPs for 3 h, washed in PBS and then resuspended in fresh medium. Between 0–60 minutes after PDT, cells were collected, washed in PBS, and incubated with DCFH–DA (25 mg/ml) for 20 min at 37 °C in the dark. The fluorescence intensity of stained cells was determined at an excitation wavelength of 488 nm and emission at 530 nm on a fluorimeter microplate reader (Bio Tek Instruments, Winooski, USA). For flow cytometric evaluation of ROS, the mean fluorescence intensity of DCF was measured at 30 min after PDT using Cyflogic v. 1.2.1.

### Cell differentiation assays

For morphological assessment of differentiation, cytospin slides of the cells were stained with Wright-Giemsa solution and observed using light microscopy (Olympus, Japan). Megakaryocytic differentiation was evaluated by staining K562 cells with a CD41 antibody conjugated with fluorescein isothiocyanate (FITC) and phycoerythrin (PE)-labeled CD61 antibodies (Dako, Glostrup, Denmark). Fluorescence signals were recorded and analyzed by Partec PAS flow cytometer (Partec GmbH, Münster, Germany). An isotope that matched the IgG was used as a control for these experiments^47^.

### Colorimetric caspase activity assay

Caspase activity was evaluated using the Apo Target™ Caspase Colorimetric Sampler Kit (Invitrogen Life Technologies; Carlsbad, CA, USA), according to the manufacturer’s instructions. Cytosolic extracts were assayed for their protein concentrations using the Bradford method before being incubated with individual substrates for each caspase (i.e., VDVAD for caspase-2, DEVD for caspase-3, VEID for caspase-6, IETD for caspase-8, and LEHD for caspase-9) labeled with para-nitroaniline (pNA). Cells were washed with PBS and lysed in Tris-HCl buffer, incubated on ice for 10 min and centrifuged for 1 min (10,000 × *g*). Protein (50 μg) per 50 μl cell lysis buffer (1 mg/ml) was used for each assay in the presence of caspase substrates (200 μM). Upon cleavage by corresponding caspases, pNA absorption was quantified at 405 nm. The results are presented as fold-increase in caspase activity.

### Annexin V/ PI apoptosis assay

Apoptosis was quantified using the annexin V-FITC and PI methods and the Dead Cell Apoptosis kit (Invitrogen Life Technologies). Control and PDT-treated cells were washed in cold PBS and diluted in annexin-binding buffer at a density of 10^6^ cells/ml. Following addition of annexin-V FITC (1 μM) and PI (1 mg/ml), cells were incubated for 10 min in the dark at RT, analyzed by Partec Pas flow cytometry^47^ and separated into 3 groups: Annexin V^−^/PI^−^ (intact live cells), Annexin V^+^/PI^−^ (early apoptotic cells) and Annexin V^+^/PI^+^ (late apoptotic cells).

### Detection of acidic vesicular organelles

Development of acidic vesicular organelles (AVOs) is a morphological feature, and also a marker, of late autophagy and was quantified by acridine orange (AO) staining^48^,^49^. AO has green fluorescence but once protonated within the acidic compartments of the cells, it emits a bright red fluorescence. Cells (5 × 10^5^/ml) were exposed to photo-activated N-TiO_2_ for 12–48 h, harvested and stained with AO (100 μg/ml) at RT for 10 min in the dark. Following washing with PBS, images were analyzed with a fluorescence microscope (Olympus, Japan). A portion of the stained cells was resuspended in 500 ml PBS and intensities of both green (FL1, 550 nm) and red (FL3, 650 nm) fluorescence was quantified with a Partec Pas Flow cytometer (Partec GmbH, Münster, Germany). The percentage ratio of FL3/FL1 positive cells (red-to-green fluorescence intensity) was considered to be an indicator of AVO formation.

### Immunocytochemistry and confocal microscopy

Cells were seeded on coverslips, exposed to different concentrations of N-TiO_2_ and 24 h after PDT cells were washed with PBS, fixed in 4% paraformaldehyde in PBS (pH 7.5), and permeabilized for 30 min with 0.5% Tween-20 in 5% BSA blocking solution. For analysis of LC3-β and LAMP2 punctuations, cells were incubated with rabbit polyclonal LC3B antibody (1:300, Cell Signaling, Cat#: 2775) and/or rat monoclonal LAMP2 antibody (1:50, Santa Cruz Biotechnology Cat#: sc-20004) overnight at 4 °C, followed by incubation with a goat anti-rabbit IgG Alexa Fluor 488 (Life Technologies, Cat# A-11070) and goat anti-rabbit IgG Alexa Fluor 546 (Life Technologies, Cat# A11081) for 1 h at RT, respectively. Images were captured using a Leica CTRMIC 6000 confocal microscope equipped with a Hamamatsu C910013 spinning disc camera (Leica Microsystems Inc., Wetzlar, Germany) and analyzed using Velocity software.

### Western blotting

Cells were lysed on ice in NaCl (150 mM), sodium deoxycholate (0.5%), SDS (0.1%), Tris-HCl (50 mM, pH 7.4), Triton X-100 (1%), EDTA (1 mM), EGTA (1 mM), NaF (1 mM), Na_3_VO_4_ (2 mM), sodium pyrophosphate (20 mM), sodium deoxycholate (0.5%), PMSF (1 mM), aprotinin (50 μg/ml), leupeptin (10 μg/ml), and pepstatin (1 μg/ml). Forty to fifty micrograms of protein was used for SDS-polyacrylamide gel electrophoresis and protein transfer onto nitrocellulose membrane (Whatman, UK). Membranes were blocked with Tris-buffered saline (pH 7.4) containing 5% fat-free milk powder and Tween-20 (0.2%) for 2–3 h at RT^47^,^81^ and incubated with the anti-beta actin (ab8227), and anti-LC3B (ab51520) monoclonal antibodies (Abcam, Cambridge, MA, USA) overnight at 4 °C. Membranes were washed 3 times in PBS-Tween-20 (0.2%) and incubated between 1–2 h at RT with appropriate horseradish peroxidase-conjugated secondary antibodies (1:10.000; Sigma). Protein bands were visualized using an enhanced chemiluminescence kit (Amersham Life Sciences, UK).

### Statistical analysis

Data are expressed as the mean ± standard deviation (SD) of at least three independent experiments, each performed in duplicate or triplicate. The statistically significant differences (P < 0.05) were analyzed using student’s t-test and ANOVA by Microsoft Excel 2013 (Microsoft, Washington, USA) and GraphPad Prism 5.03 software (La Jolla, CA, USA).

## Additional Information

**How to cite this article**: Moosavi, M. A. *et al.* Photodynamic N-TiO_2_ Nanoparticle Treatment Induces Controlled ROS-mediated Autophagy and Terminal Differentiation of Leukemia Cells. *Sci. Rep.* **6**, 34413; doi: 10.1038/srep34413 (2016).

## Supplementary Material

Supplementary Information

## Figures and Tables

**Table 1:** Dispersing parameters and cellular uptake of N-TiO_2_ NPs in cell culture condition.

Particle	Average diameter (nm)	Zeta potential (mV)	Mean SSC changes (treated/control)
N-TiO_2_ in water	773 ± 24	+1.5 ± 0.6	−
N-TiO_2_ in media	951 ± 55	−9.0 ± 1.5	5.9 ± 0.5
N-TiO_2_ (100 μg/ml) in media +10% FBS	98 ± 27	−16.3 ± 1.4	7.2 ± 0.9
N-TiO_2_ (10 μg/ml) in media +10% FBS	92 ± 12	−19.0 ± 2.1	6.1 ± 0.6

NPs were dispersed in water or RPMI media with or without fetal bovine serum (FBS; 10%, v/v), then sonicated, vortexed and hydrodynamic sizes and cell surface charges (zeta potential) were measured. For cellular uptake experiments, K562 cells were treated with N-TiO2 for 3 h in the dark, then mean fluorescent intensity (MFI) of SSC (side-scattered light) were analyzed using flow cytometry, and expressed as the fold change of control cell MFI. The results are presented as the mean of three independent replicates ± SD.
