# Supplementary material for: Photodynamic N-TiO2 Nanoparticle Treatment Induces Controlled ROS-mediated Autophagy and Terminal Differentiation of Leukemia Cells
Source: Sci Rep. 2016 Oct 4;6:34413. doi: 10.1038/srep34413 (PMC5048164; doi:10.1038/srep34413)
Supplement: Supplementary Information [file srep34413-s1.doc]

***Supplementary data of***

**Photodynamic N-TiO_2_ Nanoparticle Treatment Induces Controlled ROS-mediated Autophagy and Terminal Differentiation of Leukemia Cells**

Mohammad Amin Moosavi^1*^, Maryam Sharifi^1,2^, Soroush Moasses Ghafary^3,4^, Zahra Mohammadalipour^1^, Alireza Khataee^5,6^, Marveh Rahmati^7^, Sadaf Hajjaran^1^, Marek J. Łos^8^, Thomas Klonisch^9^, Saeid Ghavami^9-11*^

^1^Department of Molecular Medicine, Institute of Medical Biotechnology, National Institute for Genetic Engineering and Biotechnology, Tehran, Iran

^2^Cell and Molecular Biology Department, Pharmaceutical Sciences Branch, Islamic Azad University, Tehran, Iran

^3^ Hematology and Oncology Research Center, Tabriz University of Medical Science, Tabriz, Iran

^4^Department of Nanobiotechnology, Faculty of Biological Sciences, University of Tarbiat Modares, Tehran, Iran

^5^Research Laboratory of Advanced Water and Wastewater Treatment Processes, Department of Applied Chemistry, Faculty of Chemistry, University of Tabriz, Tabriz, Iran

^6^ Department of Nanotechnology, Near East University, 99138 Nicosia, North Cyprus, Mersin 10, Turkey

^7^Rheumatology Research Center, Tehran University of Medical Sciences, Tehran, Iran

^8^LinkoCare Life Sciences AB, 583 30 Linköping, Sweden; Department of Patomorphology, Pomeranian Medical University, Szczecin, Poland

^9^Department of Human Anatomy and Cell Science, College of Medicine, Faculty of Health Sciences, University of Manitoba, Winnipeg, MB, Canada

^10^Children Hospital Research Institute of Manitoba, University of Manitoba, Winnipeg, Canada

^11^Health Research Policy Centre, Shiraz University of Medical Science, Shiraz, Iran

*^#^*These authors contributed equally to this work.

***Corresponding authors:**

**1-Ghavami S,** Assistant Professor

Department of Human Anatomy and Cell Science, Max Rady College of Medicine, Rady Faculty of Health Sciences, University of Manitoba, Winnipeg, MB, R3E 3P4, Canada

Tel: +1 (204) 612-8956, Fax: (+98) 21 44787399

Email: [saeid.ghavami@umanitoba.ca](mailto:ghavami@cc.umanitoba.ca)

**2-Moosavi MA,** Assistant Professor

Department of Molecular Medicine, Institute of Medical Biotechnology, National Institute of Genetic Engineering and Biotechnology, Tehran-Karaj Highway, Tehran, Iran

P.O. Box 14965/161, Tehran, Iran

Tel/Fax: (+98) 21 44787335

Email: [a-moosavi@nigeb.ac.ir](mailto:a-moosavi@nigeb.ac.ir)

**Supplementary Figure Legends**

**Figure S1. Physicochemical characterization of N-TiO_2_ NPs dispersed in water with or without FBS.** Synthesized N-TiO_2_ NPs were dispersed in dH_2_O and sonicated for 15 min, and their morphology (aggregation state) and average hydrodynamic sizes were analyzed using SEM (A) and DLS (B), respectively. The results show aggregation/agglomeration of NPs in this condition. The average hydrodynamic sizes of NPs in water with FBS (C) were also analyzed by DLS. In this experiment, N-TiO2 NPs were dispersed in dH2O and sonicated for 15 min, then FBS (10%, v/v) were added. As can be observed, FBS reduced aggregation size of N-TiO_2_ in water.

**Figure S2. Comparing effects of N-TiO_2_ in dark and visible light condition.** The cells were exposed to 0 and 10 µg/ml well-dispersed NPs in dark and visible light condition, then the growth was estimated using the trypan blue exclusion test. For light condition, the cells were illuminated with single doses of visible light (10 min) using a 55W Xenon lamp. All the results are from four independent experiments ± SD, each performed in triplicate.

**Figure S3. Effects of different N-TiO_2_-based PDT doses on morphology and apoptosis of normal PBLs.** The cells were exposed to low (10 µg/ml, 12 J/cm^2^) and high (100 µg/ml, 12 J/cm^2^) doses of PDT for 24 h, and morphological changes were studied using light microscopy (magnification 40×). B) PBLs were exposed to 10 and µg/ml N-TiO_2_ and irradiated for 10 min, then harvested after 24 h and Annexin-V/PI double staining was measured to detect apoptosis using flow cytometry.

**Figure S4. Effects of light-irradiated N-TiO_2_ in erythroid differentiation of K562 cells.** Cells were exposed to N-TiO_2_ (10 and 100 µg/ml) and irradiated for 10 min, and hemoglobin-containing differentiated cells were observed and counted using a light microscope following benzidine staining. GTP (100 µM) was used as a positive control for erythroid differentiation. There was more than a 40% increase in number of benzidine-positive cells (light blue cells indicated by arrows) in the GTP-treated cells, but the control and N-TiO_2_ exposed cells did not show benzidine positivity.

**Figure S5. Autophagy effects of light-irradiated N-TiO_2_ in PBLs.** (A) PBLs were exposed to N-TiO_2_ (10 and 100 µg/ml) and irradiated for 10 min,and AVO formation and LC3B protein expression levels were monitored at 24 h after PDT using flow cytometry and immunoblotting, respectively. The results in A are % of FL3/FL1 intensity (mean ± SD). Statistical significance was expressed as *P<0.001 compared with control cells (0 h). In B, the actin was used as loading control of proteins and data are representative of one typical experiment.

**Figure S6. Effects of 3-MA on autophagic effects of light-irradiated N-TiO_2_ in K562 cells.** K562 cells exposed to high PDT (100 µg/ml N-TiO_2_, 12 J/cm^2^), in the presence or absence of autophagy inhibitor (3-MA, 1 mM) for 24 h, then LC3-β fragmentation was studied using western blotting. Actin was used as a loading control for protein. Representative data from one experiment are shown.

**Figure S1**


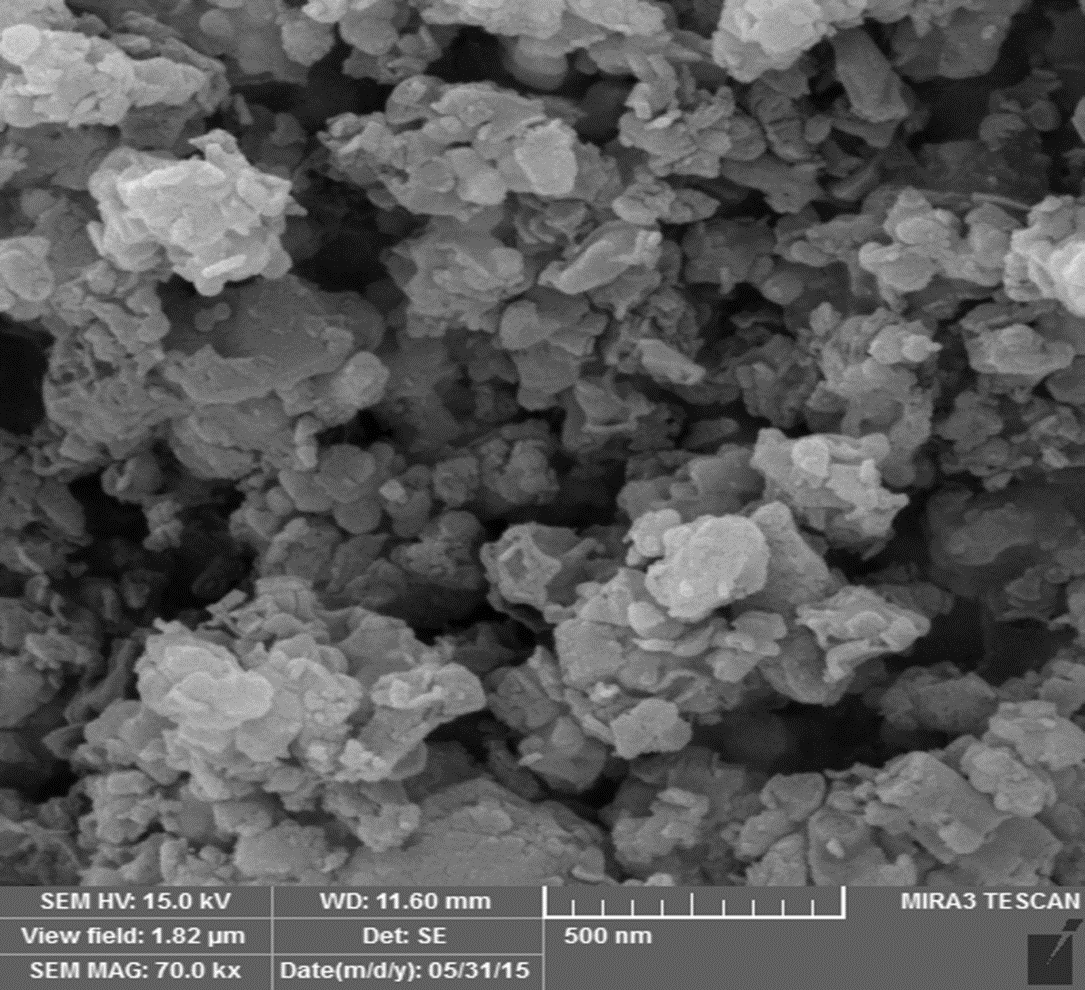


**A**

**B**


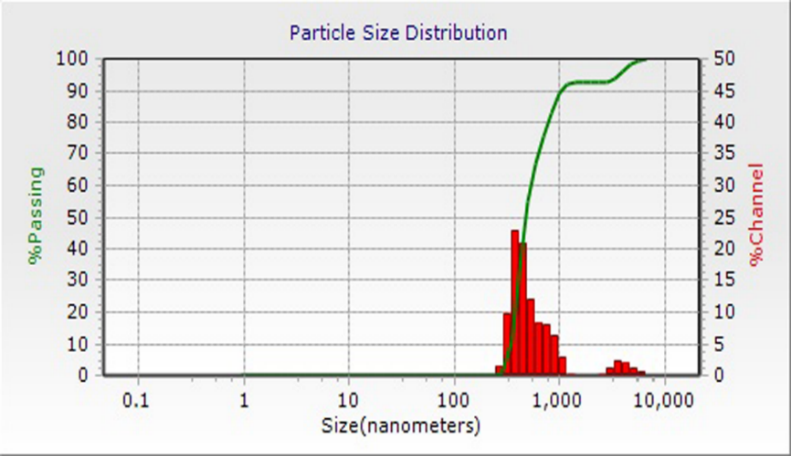


**C**


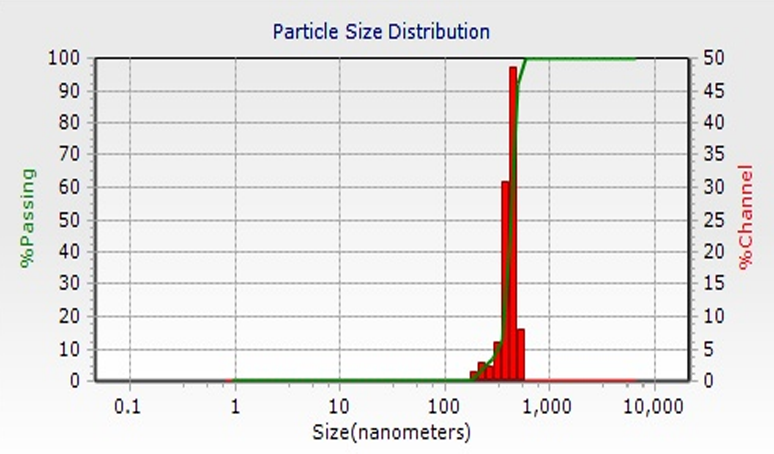


**Figure S2**

**
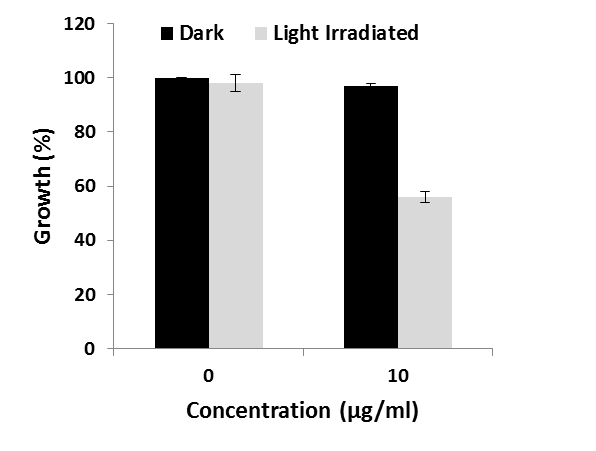
**

**Figure S3**

**
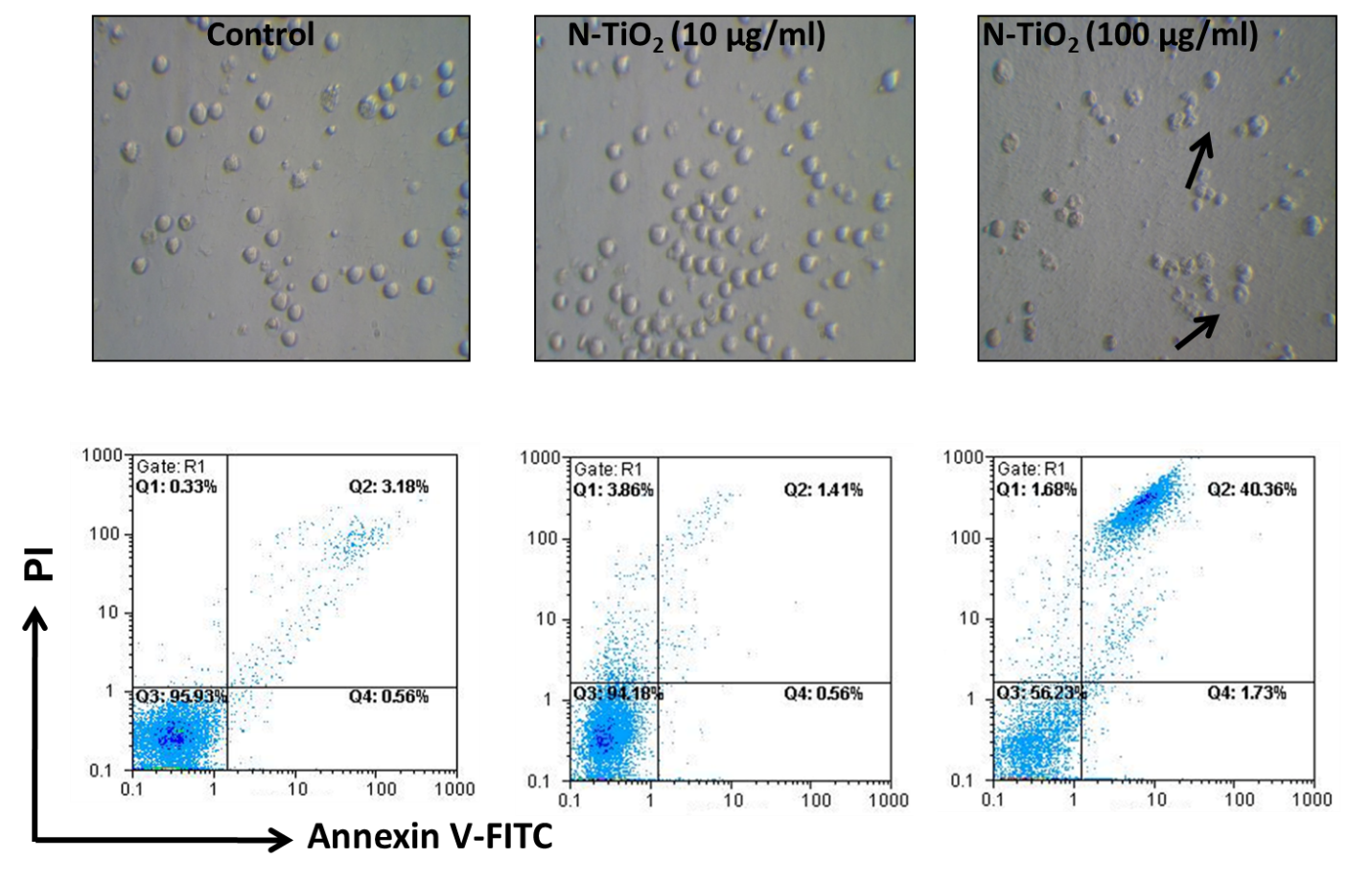
**

**Figure S4**

**
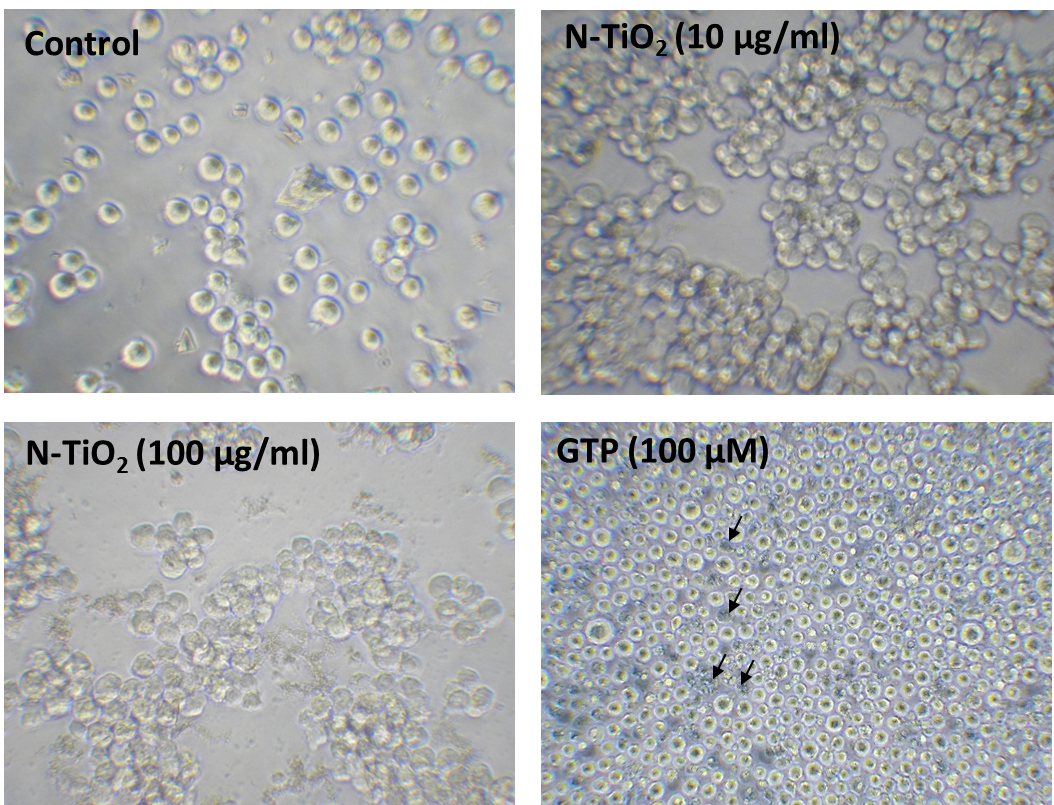
**

**Figure S5**

**
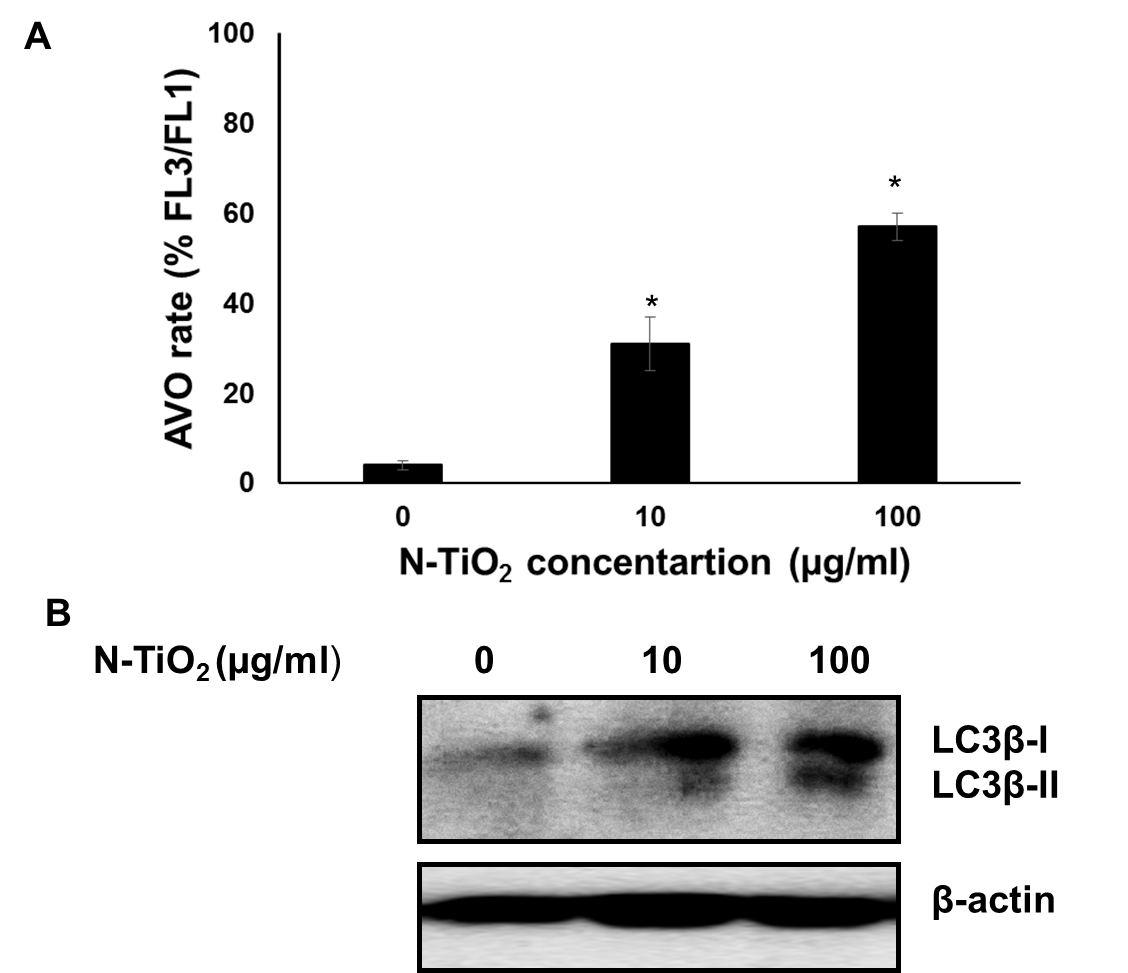
**

**Figure S6**

**
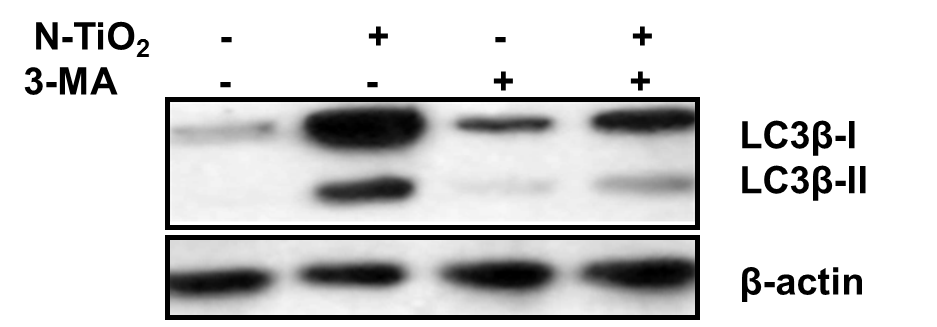
**
